# Pyrogallol-Phloroglucinol-6, 6-Bieckol Restored Primary Cilia Length, Which Was Decreased by High-Fat Diet in Visceral Adipose Tissue, and Decreased Adipogenesis

**DOI:** 10.1155/2022/8486965

**Published:** 2022-04-16

**Authors:** Seyeon Oh, Myeongjoo Son, Ji Tae Jang, Chul Hyun Park, Kuk Hui Son, Kyunghee Byun

**Affiliations:** ^1^Functional Cellular Networks Laboratory, Department of Medicine, Graduate School and Lee Gil Ya Cancer and Diabetes Institute, College of Medicine, Gachon University, Incheon 21999, Republic of Korea; ^2^Department of Anatomy & Cell Biology, Gachon University College of Medicine, Incheon 21936, Republic of Korea; ^3^Aqua Green Technology Co., Ltd., Smart Bldg., Jeju 63243, Republic of Korea; ^4^Department of Thoracic and Cardiovascular Surgery, Gachon University Gil Medical Center, Gachon University, Incheon 21565, Republic of Korea

## Abstract

Length of primary cilia, which involves cell cycle reentry and disassembly of cilia, promotes cell mitosis. It is known that the cilia length in adipose tissue of the high-fat diet (HFD) animals was shortened and accompanied by increased adipogenesis. Male C57BL/6N mice were randomly divided into groups. The mice group was given the normal fat diet (NFD/saline), HFD mice group for 4 weeks, and then HFD was also treated for the next 4 weeks with saline (HFD/saline), *Ecklonia cava* extract (HFD/ECE), or pyrogallol-phloroglucinol-6, 6-bieckol, a segment of ECE (HFD/PPB). We evaluated the effect of ECE and PPB on modulating cilia length of visceral adipose tissue and decreasing adipogenesis by decreasing cell cycle reentry using an HFD-fed mouse model. ECE and PPB decreased physiological changes, which increased by HFD, but ECE and PPB decreased the upregulation of the IL-6/STAT3/AURKA signaling pathway, which is involved in cilia disassembly. In addition, ECE or PPB elongated the cilia and decreased cyclin A2 and Cdk2 expression, which promote cell cycle reentry, and decreased the adipogenesis genes. PPB and ECE restored cilia length and decreased adipogenesis through modulating the IL-6/STAT3/AURKA pathway and decreasing cell cycle reentry in the visceral adipose tissue of HFD/saline mice group.

## 1. Introduction

Cilia are the extruding structure of eukaryotic cells. They are classified into motile and immotile cilia [1, 2]. The motile cilia are present in the respiratory tract, while immotile cilia are present in almost every cell in the body [1, 2]. Immotile cilia, which are also called primary cilia, act as a sensor and are involved in signal transduction by sensing environmental information [1, 2].

Primary cilia are also involved in regulating the cell cycle. Primary cilia are present during G0 and G1, and in S/G2, they are resorbed before entering mitosis [3]. The primary cilium's presence keeps cells in a quiescent state and prohibits cell proliferation, while cell division is initiated when the cilium is resorbed into the cell [4]. Resorption or disassembly, when cilium retracts into the cell, leads to the reentry of cells into the cell cycle by initiating mitosis [5].

White adipose tissue enlarges by both hypertrophy (increasing adipocyte size) and hyperplasia (increasing adipocyte number) [6, 7]. Differentiation of adipocyte precursor cells inherent in adipose tissue leads to adipocyte hyperplasia and this process is called adipogenesis [8]. The primary cilium is also involved in adipogenesis by regulating the cell cycle entering [9].

Mutations in proteins related to cilia formation lead to various diseases such as Bardet–Biedl syndrome or Alstrom syndrome 1. Those diseases showed primary cilium defects and were accompanied by obesity [10–12]. Several studies showed that both primary obese adipose tissue and adipose-derived mesenchymal stem cells (ASCs) from obese human subjects showed shorter cilia than lean control subjects [13]. It is known that a high-fat diet (HFD) leads to the defect of primary cilia and enhances cell cycle reentry at an early stage of adipogenesis, which increases adipogenesis [14].

In obesity, the expansion of adipose tissue leads to chronic inflammation and hypoxia in the adipose tissue, and preadipocytes and adipocytes are often exposed to inflammatory cytokines such as interleukin (IL)-6 or tumor necrosis factor-alpha (TNF-*α*) [15, 16]. TNF-*α* and IL-6 are known to involve in the shortening of cilia in ASC [13]. IL-6 regulates various downstream genes by activating the signal transducer and activator of transcription (STAT) 3 [17]. One downstream of IL-6 is aurora kinase A (AURKA), a vital regulator of cilium disassembly [18]. AURKA is directly regulated by the IL-6/Janus kinase 2 (JAK2)/STAT3 pathway via c-myc [19]. TNF-*α* also initiates the upregulation of the JAK1/STAT3/STAT5 pathway [20]. The mitotic kinases polo-like kinase 1 (Plk1) and AURKA are crucial during cilia's disassembly process [21]. By mitogen stimulation during the early G1 phase, Plk1 inhibits the extension of the axoneme via activation of kinesin family member 2A (Kif2A), which is a potent microtubule depolymerase [21]. Plk1 cooperates with AURKA in the disassembly process of primary cilia [22]. Furthermore, activation of kinesin family member 24 (Kif24) ensures ciliary disassembly from the S phase of mitosis [23]. AURKA is increased in ASCs of obese adipose tissue, and it was increased in the ASCs of lean adipose tissue by treatment of IL-6 and TNF-*α* [18]. Inhibition of AURKA restores the cilium length of obese ASC and IL-6-treated lean ASCs [18]. Those results suggested that AURKA is a major downstream signal, which was upregulated by IL-6 [18].

*Ecklonia cava* is a brown alga species abundant in Korea and Japan [24]. Previously, our group reported that *E. cava* extract (ECE) decreased leptin resistance in adipose tissue induced by HFD via decreasing inflammation of adipose tissue [25]. Pyrogallol-phloroglucinol-6, 6-bieckol (PPB), one segment of ECE, is known that has an anti-obesity effect by decreasing the inflammation of the fat tissue in the HFD-fed animal model [26]. Previous studies showed that ECE or PPB attenuated obesity by decreasing inflammation in the adipose tissue. It has not been revealed whether ECE or PPB affects cilia length modulation, which is related to adipogenesis by decreasing adipose tissue inflammation [25, 26]. In the present study, we evaluated whether ECE or PPB decreased IL-6, which eventually led to a decrease in the signal pathway of JAK2/STAT3/AURKA related to cilia disassembly in the visceral adipose tissue of HFD-fed animals. The effects of ECE and PPB on restoring cilia length of adipocyte, which was accompanied by decreasing signals related to cilia shortening such as Plk1, Plk4, Kif2A, and Kif24, were also evaluated. We also evaluated whether ECE and PPB decreased proteins that enhance cell cycle entry such as cyclin A2 and cyclin-dependent kinase 2 (Cdk2), which eventually attenuated adipogenesis in the visceral adipose.

## 2. Materials and Methods

### 2.1. Prepared ECE and PPB from *E. cava*

The extract was obtained from the Aqua Green Technology Co., Ltd. (Jeju, Republic of Korea). Briefly, the raw materials of *E. cava,* washed with pure water, were air-dried at ambient temperature for 48 hours and finely ground, and 50% ethanol was added and heated at 85 degrees for 12 hours. Thereafter, the ECE was subjected to filtration and concentration, heat sterilized at high temperature for one hour, and then spray-dried [25–28].

The PPB, one of the four phlorotannins of *E. cava*, was isolated in the same method as in the previous studies [25–28]. Briefly, a column was charged with an organic stationary phase using centrifugal partition chromatography in a two-phase solvent system mixing distilled water, ethyl acetate, methanol, and mixture of n-hexane (ratio 7:7:3:2). The mobile phase was charged to the column in descending order (flow rate of 2 mL/min) and separated. The purity of the isolated PPB was measured to be about 91.24% [25–28] and used in this study.

### 2.2. HFD-Fed Mice Model

Animal experiments in this study were conducted in accordance with the ethical principles announced by the Institutional Animal Care and Use Committee of Gachon University (approval number: LCDI-2019-0130). The 7-week-old male C57BL/6N mice were purchased from Orient Bio (Seongnam, Republic of Korea) and they were bred while maintaining a constant temperature of 22°C to 23°C, relative humidity of 45% to 50%, and dark/light cycle at 12-hour intervals. After one week of adaption, the mice were haphazardly assigned to six groups as follows: a group ingested a regular normal fat diet (NFD) for 4 weeks and followed by oral administration of 0.9% normal saline with NFD intake for 4 weeks (1, NFD/saline). After consuming a 45% HFDs (Research diet Inc., NY, USA) for 4 weeks, 0.9% saline (2, HFD/saline), 50, 100, and 150 mg/kg/day of ECE (3–5, HFD/ECE 50, 100, and 150) or 2.5 mg/kg/day of PPB (6, HFD/PPB) by oral administration with a 45% HFD for the next 4 weeks. Eight weeks after the start of treatment, all mice were measured for mice body weight and fat mass by Minispec MQ Series (Bruker, MA, USA) before sacrifice [28, 29].

### 2.3. Adipocyte Size Measure

To measure the size of adipocytes in visceral fat, the adipose tissue slides were stained with hematoxylin & eosin. The adipose tissue slides were deparaffinized with xylene and then rehydrated using progressive ethanol solution (100%, 90%, 80%, and 70%) for 1 minute each step. After rinsing the slides with running water, the slides were immersed in hematoxylin solution and washed with tap water for 3 minutes. The tissue slides were immersed into the eosin solution for 1 minute and washed with running water. Cover slides were mounted using xylene-based DPX solution (Sigma-Aldrich, MO, USA) and visualized with an optical microscope (BX53M; Olympus, Japan). The adipocytes size of visceral fat tissue was determined by randomly capturing 10 visceral fat images and the adipocyte area was measured from the cross-sectional area of the adipocyte membrane by the Image J software (NIH, DC, USA) [28, 29].

### 2.4. Western Blot for Primary Cilia

The frozen visceral fat was homogenized with the RIPA buffer (EzRIPA, ATTO, Tokyo, Japan) containing phosphatase and proteinase inhibitors. The lysed adipose tissues were subjected to sonication and then centrifuged at 4°C for 15 minutes at a speed of 14,000 × *g*. For the separation of the equal amount of extracted visceral fat proteins, it was loaded on 8–12% polyacrylamide gels using electrophoresis (Bio-Rad Laboratories, CA, USA) and transferred onto polyvinylidene fluoride membranes (Millipore, MA, USA) by a PowerStation (ATTO). After blocking using 5% skim milk for 1 hour at room temperature, the membrane was washed with Tris-buffered saline containing 0.1% Tween 20 (TTBS). The primary antibodies listed Supplementary Table 1 (Table S1) were incubated at 4°C for overnight. After washing the membrane tagged with the primary antibodies with TTBS, it was incubated with horseradish peroxidase-conjugated secondary antibodies (Vector Laboratories, CA, USA) and rinsed again using TTBS. Subsequently, it was developed using a chemiluminescence detection solution (GE Healthcare, IL, USA) to visualize the proteins on the membrane. Western blot images obtained through visualization were analysed using the Image J software (NIH, DC, USA) [30, 31].

### 2.5. Immunofluorescence for Primary Cilia

For tissue staining, the visceral adipose tissue paraffin blocks, sectioned to a thickness of 7 *μ*m, were placed on gelatin-coated slides and dried at 37°C for overnight. The adipose tissue placed on the coated slides was deparaffinized with xylene and progressive ethanol (100%, 90%, 80%, and 70%) and then washed three times with phosphate-buffered saline (PBS). After that, we removed the PBS and the tissue slides incubated with animal serum for antigen-antibody blocking and anti-Arl13b primary antibody (Proteintech; IL, USA) and rinsed it with PBS. The tissues were loaded for 1 hour at room temperature with Alexa Fluor 488 (Invitrogen; MA, USA) and then washed with PBS. Finally, tissue slides washed with PBS were incubated with a nucleus staining solution (4′, 6-diamidino-2-phenylindole; DAPI) for 5 minutes and then removed using PBS. After that, the slides were prepared for confocal imaging using vector shield solution (Vector Laboratories, CA, USA). The Arl13b positive cilia were visualized and analysed using a confocal microscope (LSM 710, Carl Zeiss, Oberkochen, Germany) at the core facility for cell-to-in vivo imaging. At least 20 confocal images of the Arl13b positive cilia were randomly captured. Cilia length measurements in visceral fat tissue were determined by drawing a line across the cilia in the proximal or distal region using the Image J software (NIH, DC, USA) [32, 33] using Arl13b as the cilium marker [13].

### 2.6. Quantitative Real-Time Polymerase Chain Reaction (qRT-PCR)

The visceral adipose tissue finely pulverized using liquid nitrogen was incubated with 1 mL of RNAiso Plus reagent (TAKARA, Shiga, Japan) at room temperature for 5 minutes. The lysed samples were centrifuged at a speed of 12,000 × *g* at 4°C for 5 minutes, and then the supernatant is collected and transferred to a new tube. The transferred supernatants were vigorously mixed with 0.2 mL of chloroform (Amresco, OH, USA), incubated at room temperature for 5 minutes, and then centrifuged at 12,000 × *g* for 15 minutes at 4°C. At this time, the collected aqueous phase was mixed with 0.25 mL of isopropanol, placed at room temperature for 10 minutes, and then centrifuged at 12,000 × *g* for 10 minutes at 4°C again. The extracted RNA pellets were thoroughly washed with cold 75% ethanol and centrifuged at 7,500 × *g* at 4°C for 5 minutes, and then the supernatant was discarded to dry the RNA pellet. The dried pellet was dissolved using 20–30 *μ*L of diethylpyrocarbonate (DEPC)-treated water, and the concentration and quality of the extract RNA were measured by a Nanodrop 2000 (Thermo Fisher Scientific, MA, USA). For qRT-PCR, the extracted RNA was synthesized as complementary DNA (cDNA) using a cDNA synthesis kit (PrimeScript™, TAKARA) according to the protocol provided by the manufacturer. To determine the RNA level, qRT-PCR was performed, and the primers (listed in Table S2) used were mixed with DEPC-treated water and placed in a 384-well plate. After that, the cDNA template and SYBR green reagent (TAKARA) were further aliquoted and then verified by the qRT-PCR instrument (Bio-Rad, CA, USA).

### 2.7. Statistical Analysis

In order to verify the significance of the differences between animals, the D'Agostino & Pearson omnibus normality test was conducted, and then 6 groups were compared by the Kruskal–Wallis test, followed by a post hoc test using Mann–Whitney *U* test. The dam study was verified using an unpaired *t*-test. All results are expressed as mean ± standard deviation, and the statistical significance was represented as follows: ^*∗*^, vs. NFD/saline; ^$^, vs. HFD/saline; ^#^, vs. HFD/ECE100; ^†^, vs. HFD/PPB. The statistical analyses used in this study were determined by SPSS statistics 20 software (IBM Corporation, Armonk, NY, USA).

## 3. Results

### 3.1. ECE and PPB Reduced Body Weight, Fat Weight, and Adipocyte Size of the Visceral Adipose Tissue of HFD Animals

**Figure 1:**
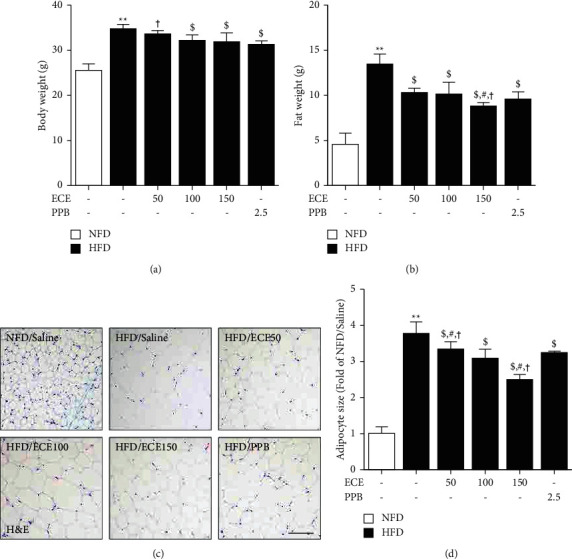
ECE and PPB decreased body weight, fat weight, and visceral adipocyte size in HFD animals. C57BL/6N male mice were fed a regular NFD or HFD for 4 weeks, and then NFD or HFD were also treated for the next 4 weeks with various substances. (a) Body weight; (b) fat weight increased in HFD mice, but ECE and PPB reduced; ((c), (d)) H&E stained visceral adipocyte size also decreased in ECE and PPB administrative mice and measured by using the Image J software. Scale bar = 100 *μ*m. All data are shown as mean ± S.D. ^*∗∗*^, *p* < 0.01, vs. NFD/saline; ^$^*p* < 0.05, vs. HFD/saline; ^#^*p* < 0.05, vs. HFD/ECE100; ^†^*p* < 0.01, vs. HFD/PPB (Mann–Whitney *U* test). ECE, *Ecklonia cava* extract; H&E, hematoxylin & eosin, HFD, high-fat diet; NFD, normal fat diet; PPB, pyrogallol-phloroglucinol-6, 6-bieckol.

The body weight of HFD animals was significantly higher than that of NFD animals (Figure 1(a)). There was a significant decrease in the HFD/ECE100, ECE150, or PPB groups. The fat weights of HFD animals were significantly higher than those of NFD animals (Figure 1(b)). It showed a significant decrease in the HFD/ECE50, ECE100, ECE150, or PPB. The most prominent decreasing effect was shown at the HFD/ECE150. The adipocyte sizes of HFD animals were significantly higher than those of NFD animals (Figures 1(c) and 1(d)). It showed a significant decrease in the HFD/ECE50, ECE100, ECE150, or PPB. The most prominent decreasing effect was shown at HFD/ECE150.

### 3.2. ECE and PPB Restore the Upregulation of IL-6/JAK2/STAT3/AURKA in the Visceral Adipose Tissue of HFD Animals

**Figure 2:**
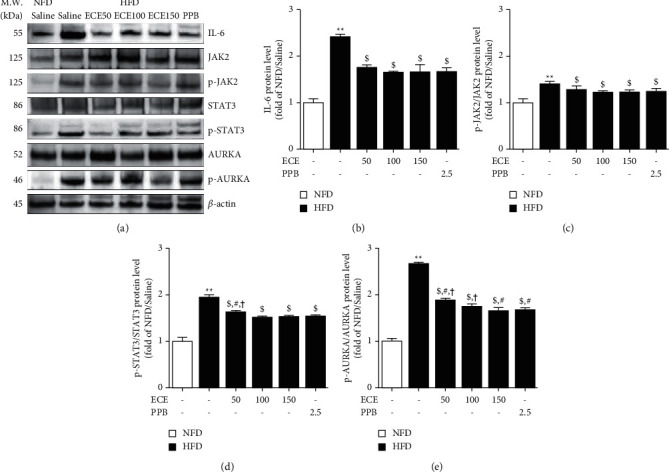
ECE and PPB restore upregulation of IL-6/JAK2/STAT3/AURKA in visceral adipose tissue of HFD animals. (a) The protein levels of IL-6/JAK2/STAT3/AURKA in visceral adipose tissue of HFD mice were assessed by Western blot. (b–e) Quantitative graphs of representative Western blot images. The protein levels were normalized to *β*-actin. Increased protein levels were restored by ECE and PPB administration. All data are shown as mean ± S.D. ^*∗∗*^, *p* < 0.01, vs. NFD/saline; ^$^*p* < 0.05, vs. HFD/saline; ^#^*p* < 0.05, vs. HFD/ECE100; ^†^*p* < 0.01, vs. HFD/PPB (Mann–Whitney *U* test). AURKA, aurora kinase A; ECE, *Ecklonia cava* extract; IL-6, interleukin 6; JAK2, janus kinase 2; HFD, high-fat diet; NFD, normal fat diet; p-AURKA, phosphorylated AURKA; p-JAK2, phosphorylated JAK2; p-STAT3, phosphorylated signal transducer and transcription 3; PPB, pyrogallol-phloroglucinol-6, 6-bieckol; STAT3, signal transducer and transcription 3.

The expression of IL-6 in the adipose tissue of HFD animals showed a significant increase than that of NFD animals (Figures 2(a) and 2(c)). It showed a significant decrease in the HFD/ECE50, ECE100, ECE150, or PPB. There was no significance among the HFD/ECE50, ECE100, ECE150, or PPB groups.

The expression ratio of phosphorylated JAK2 and total JAK2 (p-JAK2/JAK2) in the adipose tissue of HFD animals showed a significant increase than that of NFD animals (Figures 2(a) and 2(c)). It showed a significant decrease in the HFD/ECE50, ECE100, ECE150, or PPB. There was no significance among the HFD/ECE50, ECE100, ECE150, or PPB.

The expression of p-STAT3/STAT3 in the visceral adipose tissue of HFD animals showed a significant increase than that of NFD animals (Figures 2(a) and 2(d)). It showed a significant decrease in the HFD/ECE50, ECE100, ECE150, or PPB. There was no significance among the HFD/ECE100, ECE150, and PPB.

The expression of p-AURKA/AURKA in HFD animals' visceral adipose tissue showed a significant increase than that of NFD animals (Figures 2(a) and 2(e)). It showed a significant decrease in the HFD/ECE50, ECE100, ECE150, or PPB.

### 3.3. ECE and PPB Restored the Cilia Length of Adipocytes, Which Was Decreased by HFD in the Visceral Adipose Tissue

**Figure 3:**
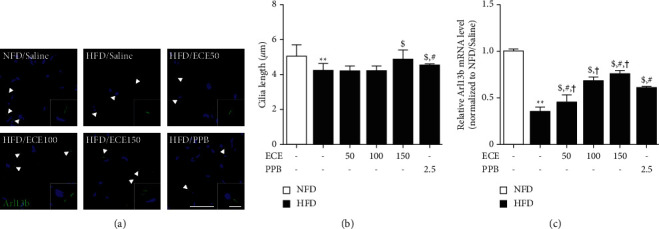
ECE and PPB restore the cilia length of visceral adipocytes in HFD animals. (a) The fluorescence images show Arl13b positive cilia (green) and nuclei (DAPI; blue) in the visceral adipocytes and (b) cilia length was measured by zen 2012 software (Zeiss microscopy image software). White arrows indicate cilia in the adipocyte and large magnification images show single cilia. Scale bar = 50 *μ*m. (c) The mRNA levels of Arl13b in visceral adipose tissue of HFD mice were measured by qRT-PCR. All data are shown as mean ± S.D. ^*∗∗*^, *p* < 0.01, vs. NFD/saline; ^$^*p* < 0.05, vs. HFD/saline; ^#^*p* < 0.05, vs. HFD/ECE100; ^†^*p* < 0.01, vs. HFD/PPB (Mann–Whitney *U* test). Arl13b, ADP-ribosylation factor-like protein 13B; ECE, *Ecklonia cava* extract; HFD, high-fat diet; NFD, normal fat diet; PPB, pyrogallol-phloroglucinol-6, 6-bieckol.

Like the previous study, the cilium length was measured using ADP-ribosylation factor-like protein 13B (Arl13b) staining as the cilium marker [13]. The cilium length of adipocytes in the visceral adipose tissue of HFD animals was significantly shorter than that of NFD animals (Figures 3(a) and 3(b)). It showed a significant increase in the HFD/ECE150 or PPB. The mRNA expression of Arl13b showed a significant decrease in the visceral adipose tissue of HFD animals than that of NFD animals (Figure 3(c)). It showed a significant increase in the HFD/ECE50, ECE100, ECE150, or PPB. The most prominent increasing effect was shown at HFD/ECE150 group.

### 3.4. ECE and PPB Reduced the Signal Pathway, Which Induced Decreasing Cilia Length

**Figure 4:**
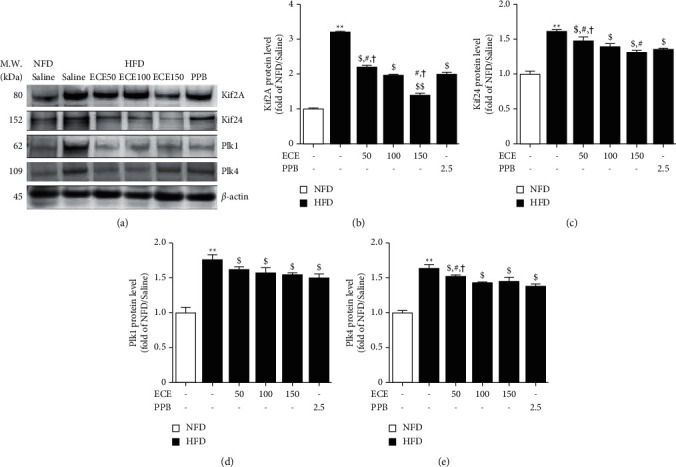
ECE and PPB modulated the cilia pathway in visceral adipose tissue of mice fed HFD. (a) The protein expression levels of Kif2A, Kif24, Plk1, and Plk4 in visceral adipose tissue of mice fed HFD were measured by Western blot. ((b)–(e)) Quantitative graphs of representative Western blot images. The protein expression levels were normalized to *β*-actin. Increased protein expression levels were restored by ECE and PPB administration. All data are shown as mean ± S.D. ^*∗∗*^, *p* < 0.01, vs. NFD/saline; ^$^*p* < 0.05 and ^$$^*p* < 0.01, vs. HFD/saline; ^#^*p* < 0.05, vs. HFD/ECE100; ^†^*p* < 0.01, vs. HFD/PPB (Mann–Whitney *U* test). ECE, *ecklonia cava* extract; HFD, high-fat diet; Kif2A, kinesin family member 2A; Kif24, kinesin family member 24; NFD, normal fat diet; Plk1, Polo-like kinase 1; Plk4; polo-like kinase 4, PPB, pyrogal-lol-phloroglucinol-6, 6-bieckol.

Plk1 and Plk4 are mitotic kinase genes, and Kif2A and Kif24 are two depolymerase genes, which are crucial regulators for both mitosis and deciliation [18, 34]. The expressions of Kif2A and Kif24 showed a significant increase in visceral adipose tissue of HFD animals than that of NFD animals (Figures 4(a)–4(c)). Those showed a significant reduction in the HFD/ECE50, ECE100, ECE150, or PPB. The decreasing effects on Kif2A and Kif24 were most prominent at HFD/ECE150 group. The expressions of Plk1 and Plk4 showed a significant increase in visceral adipose tissue of HFD animals than that of HFD animals (Figures 4(a), 4(c), and 4(d)). Those showed a significant decrease in the HFD/ECE50, ECE100, ECE150, or PPB. The decreasing effects on Plk4 were most prominent at HFD/ECE100, ECE150, or PPB group (Figures 4(a) and 4(e)).

### 3.5. ECE and PPB Reduced Cell Cycle Reentry and Adipogenesis in the Visceral Fat of HFD-Fed Animals

**Figure 5:**
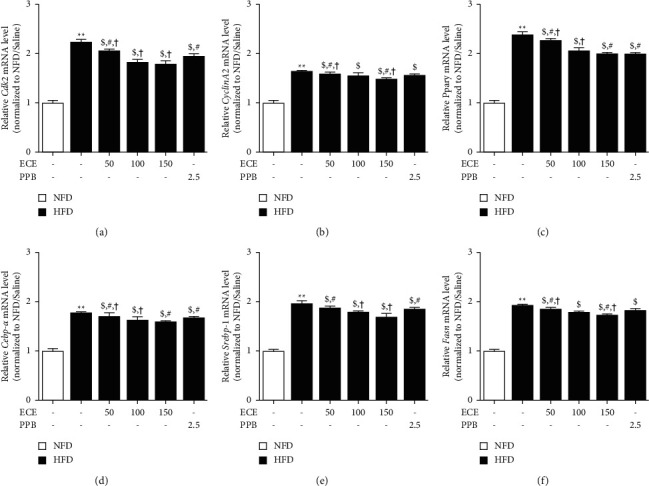
ECE and PPB reduce cell cycle reentry and adipogenesis in visceral adipose tissue of HFD mice with saline. The mRNA levels of (a) Cdk2, (b) cyclin A2, (c) ppar*γ*, (d) cebp-*α*, (e) srebp-1, and (f) fasn in visceral adipose tissue of HFD mice measured by qRT-PCR. All results are shown as mean ± S.D. ^*∗∗*^, *p* < 0.01, vs. NFD/saline; ^$^*p* < 0.05, vs. HFD/saline; ^#^*p* < 0.05, vs. HFD/ECE100; ^†^*p* < 0.01, vs. HFD/PPB (Mann–Whitney *U* test). Cdk2, cyclin-dependent kinase 2; cebp-*α*, CCAAT-enhancer binding protein alpha; ECE, *Ecklonia cava* extract; fasn, fatty acid synthase; HFD, high-fat diet; NFD, normal fat diet; ppar*γ*, proliferator-activated receptor gamma; PPB, pyrogal-lol-phloroglucinol-6, 6-bieckol; Srebp-1, sterol regulatory element-binding protein-1.

Cyclin A2 and Cdk2 are the main proteins to promote cell cycle entry [9]. HFD showed a significant increase in the expression of Cdk2 in the adipose tissue, and it showed a significant decrease in the HFD/ECE or PPB groups (Figure 5(a)). The most prominent decreasing effect was shown at HFD/ECE100 and ECE150 group. HFD showed a significant increase in the expression of Cyclin A2, and it showed a significant decrease in the HFD/ECE or PPB groups (Figure 5(b)). The most prominent decreasing effect was shown at HFD/ECE150 group. We evaluated adipogenesis with mRNA expressions of peroxisome proliferator-activated receptor gamma (Ppar*γ*), CCAAT enhancer-binding protein alpha (Cebp-*α*), sterol regulatory element-binding protein-1 (Srebp-1), and fatty acid synthase (Fasn), which are frequently used as adipogenesis markers [9]. The expression of Ppar*γ* showed a significant increase in the adipose tissue of HFD/saline, and it showed a significant decrease in the HFD/ECE or PPB (Figure 5(c)). The most prominent decreasing effect was shown at HFD/ECE150 and PPB groups. The expression of Cebp-*α* showed a significant increase in the adipose tissue of HFD/saline group, and it showed a significant decrease by treatment of ECE or PPB (Figure 5(d)). The most prominent decreasing effect was shown at HFD/ECE100 and ECE150 groups. The expression of Srebp-1 showed a significant increase in the adipose tissue of HFD/saline group, and it showed a significant decrease in the HFD/ECE or PPB groups (Figure 5(e)). The most prominent decreasing effect was shown at HFD/ECE150. The expression of Fasn showed a significant increase in the adipose tissue of HFD animals, and it showed a significant decrease in the HFD/ECE or PPB groups (Figure 5(f)). The most prominent decreasing effect was shown at HFD/ECE150 group.

## 4. Discussion

Energy access induced by calorie overload or overeating leads to enlargement of already mature adipocytes (hypertrophy) and storage of more fat [35]. Hypertrophy progresses until the adipocyte size reaches the limit of fat storage, and then hypertrophy stimulates a process of increasing adipocyte number [36]. Enlarged adipocytes secrete various hormones and cytokines to increase the recruitment of preadipocytes and the differentiation of preadipocytes into mature adipocytes [37].

Adipocyte hypertrophy is a typical finding in overweight or obesity, while adipocyte hyperplasia likely occurs at a later stage of obesity and rather represents the severity of obesity [38, 39]. In our study, the body weight and fat weight showed a significant increase by HFD, and those showed a significant decrease by administration of ECE or PPB. HFD showed a significant increase the adipocyte size in the visceral adipose tissue, and it showed a significant decrease by administration of ECE or PPB. Adipocyte hypertrophy is related to changes in biological pathways such as hypoxia, inflammation, and angiogenesis and leads to adipose tissue dysfunction [40]. Hypertrophic adipose tissue secretes inflammatory cytokines such as IL-6 and TNF-*α*, which involve the pathophysiology of metabolic diseases like increased insulin resistance [41]. IL-6 and TNF-*α* are also related to the control of cilia length [13]. It is known that obesity induced shortening of primary cilia in ASCs [13]. ASCs from obese female subjects who had a body mass index (BMI) of over 35 kg/m^2^ showed shorter cilia length, which decreased around 36% than lean control subjects with BMI of less than 24 kg/m^2^ [13].

When TNF-*α* was treated in the ASCs from lean control subjects, the cilia length was shortened [13]. Furthermore, treating IL-6 to ASCs from lean control subjects shortened cilia length [13]. Cilia length shortening by treating TNF-*α* and IL-6 in ASCs from lean subjects is accompanied by increased expressions of AURKA, Plk1, Plk4, and Kif2A [13]. It is known that the AURKA gene is upregulated by c-myc via IL-6/JAK2/STAT3 signaling pathway [19]. When MLN8054 inhibited AURKA, a small-molecule inhibitor of AURKA, the ciliary length of obese ASCs was restored [13]. Cilia length of obese ASCs by treating MLN8054 was increased by 51.9% compared with nontreated obese ASCs [13]. MLN8054 also restored the cilia length of IL-6-treated lean ASCs [13]. Thus, those results suggested that AURKA is a major effector that shortens cilia through IL-6, which prominently secretes in the obese adipose tissue. In our study, the IL-6 expression in the visceral fat tissue from animals fed HFD was significantly upregulated than that of NFD-fed animals, which showed a significant decrease by oral administration of both ECE and PPB. Moreover, the downstream processes of IL-6, JAK2/STAT3/AURKA were significantly upregulated in the visceral adipose tissue of HFD-fed animals than that of NFD-fed animals. Those signal pathways were significantly downregulated by oral treatment of ECE and PPB.

In our study, the cilia length of visceral adipose tissue of HFD-fed animals was significantly shorter than that of NFD-fed animals. The decreased cilia length was significantly restored by oral treatment of ECE or PPB. Moreover, the Arl13b expression in the visceral adipose tissue of HFD-fed animals was significantly lower than that of NFD-fed animals. It is known that the absence of Arl13b protein induced decreased cilia length and structural defects in the ciliary axoneme [42]. Like a previous study [9], this study also confirmed that HFD significantly upregulated the expression of Kif2A, Kif24, Plk1, and Plk4, and that were significantly downregulated by oral administration of ECE and PPB, which was accompanied by the shortening of cilia length.

AURKA is one of the mitotic kinases [43, 44]. When the catalytic activity of AURKA is increased, the histone deacetylase-6 (HDAC-6) phosphorylation is induced, which results in upregulation of HDAC-6-dependent tubulin deacetylation and destabilization of the ciliary axoneme [5, 45]. AURKA suppresses the primary cilia assembly, affecting G1 progression and knockdown of AURKA-induced G0/G1 arrest [45]. Thus, AURKA has an essential role in controlling cell cycle reentry by modulation of cilia length.

A previous study showed that primary cilia involved in adipogenesis with mouse stromal vascular fractions (SVFs) cultures from visceral adipose tissue [9]. The primary cilium was evaluated at four main adipocyte differentiation stages: subconfluent dividing preadipocytes, confluent preadipocytes, differentiating preadipocytes, and mature adipocytes filled with lipid droplets [9]. Cilia were not detected, or very short cilia were detected in the subconfluent dividing preadipocytes, and it was elongated after confluence the preadipocyte [9]. Cilia length was reached longest at the early stage of adipogenesis and then progressively got shortened by adipogenesis. Finally, it was almost disappeared in mature adipocytes [9].

Preadipocytes, in which cilia were pharmacologically removed with chloral hydrate, stayed at S and G2/M phases [9]. Chloral hydrate-treated SVFs showed increased cyclin A2 and CDK2, which were the main proteins for promoting cell cycle entry [9]. Moreover, disrupted cilia by knockdown Kif3a and Ifg88 increased cell number in G2/M by promoting cell cycle reentry and expression of cyclin A2 and CDK2 proteins [9]. HFD induced cilia length shortening and increased adipogenic genes' (Ppar*γ*, Cebp-*α*, Srebp-1, and Fasn) transcription in visceral SVFs, accompanied by increased cyclin A2 and CDK2 protein [9]. In our study, the expression of CDK2 and cyclin A2 showed a significant increase in the visceral adipose tissue of HFD-fed animals, and those showed a significant decrease by administration of ECE or PPB. The expressions of adipogenesis genes' (Ppar*γ*, Cebp-*α*, Srebp-1, and Fasn) showed a significant increase in the visceral adipose tissue of HFD-fed animals, and those showed a significant decrease by administration of ECE or PPB (Figure 5).

Previously, our group showed that ECE or PPB decreased inflammation in the adipose tissue, accompanied by decreased expression of IL-6 or TNF-*α* [25, 26]. Thus, ECE or PPB showed decreased body weight and fat deposition and adipose tissue dysfunction [25, 26]. However, it has not been evaluated whether ECE or PPB modulated cilia length related to cell cycle reentry and adipogenesis. In the present study, our results showed that ECE or PPB could restore cilia length, which was decreased by HFD. The signal pathway involved in cilia length restoring is probably IL-6/JAK2/STAT3/AURKA. Accompanied by increasing this pathway, Kif2a, Kif24, Plk1, and Plk4 involved in cilia disassembly were increased by HFD. As cilia length decreased, the expression of genes related to cell cycle reentry and adipogenesis increased. ECE and PPB decreased the expression of adipogenesis genes. Those results suggested that ECE or PPB could decrease adipogenesis by modulating cilia length. In addition to ECE or PPB's well-known function, which decreased inflammation in adipose tissue, which involves adipose tissue dysfunction, ECE or PPB decreased adipogenesis. Thus, ECE or PPB seems to have the potential to use as a treatment for obesity.

## 5. Conclusions

**Figure 6:**
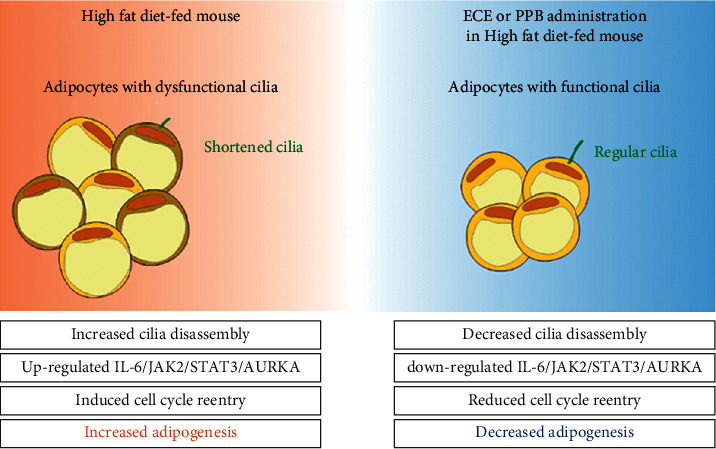
Graphical summary proposed scheme for the mechanism illustrating the regulation of primary cilia by ECE or PPB in a high-fat diet-fed mice.

In the study, PPB and ECE restored cilia length and decreased adipogenesis through modulating the IL-6/JAK2/STAT3/AURKA pathway in the visceral adipose tissue of HFD-fed animals (Figure 6).

## Data Availability

All data are contained within the article.

## Supplementary Materials

Supplementary Materials Table S1. List of antibodies for Western blot. Table S2. List of primers for quantitative real-time polymerase chain reaction (qRT-PCR).
