# Supplementary material for: Pyrogallol-Phloroglucinol-6, 6-Bieckol Restored Primary Cilia Length, Which Was Decreased by High-Fat Diet in Visceral Adipose Tissue, and Decreased Adipogenesis
Source: Int J Endocrinol. 2022 Apr 16;2022:8486965. doi: 10.1155/2022/8486965 (PMC9034920; doi:10.1155/2022/8486965)
Supplement: Supplementary Materials — Table S1. List of antibodies for Western blot. Table S2. List of primers for quantitative real-time polymerase chain reaction (qRT-PCR). [file 8486965.f1.docx]

**Supplementary material**

| **Antigen (host)** | **Company** | **Catalog no.** | **Dilution rate** |
| --- | --- | --- | --- |
| IL-6 (Rabbit) | Abcam | ab6672 | 1:500 |
| JAK2 (Rabbit) | Cell signaling technology | 3230s | 1:1,000 |
| p-JAK2 (Rabbit) | Cell signaling technology | 3776s | 1:1,000 |
| STAT3 (Mouse) | Cell signaling technology | 9139s | 1:1,000 |
| p-STAT3 (Mouse) | Santa cruz biotechnology | sc-8059 | 1:200 |
| AURKA (Mouse) | Proteintech | 66757-1-lg | 1:1,000 |
| p-AURKA (Rabbit) | GeneTex | GTX55002 | 1:500 |
| Kif2A (Mouse) | Santa cruz biotechnology | sc-271471 | 1:100 |
| Kif24 (Rabbit) | LSBio | LC-C322448-200 | 1:100 |
| Plk1 (Rabbit) | Cell signaling technology | 4535 | 1:500 |
| Plk4 (Rabbit) | Abcam | ab137398 | 1:500 |
| β-actin (Rabbit) | Cell signaling technology | 4967s | 1:1,000 |

**Table S1. List of antibodies for western blot**

**Table S2.** **List of primers for quantitative real-time polymerase chain reaction (qRT-PCR)**

| Gene name | | Primer sequence |
| --- | --- | --- |
| *β-actin* | Forward | 5'-ACA AAG CTG TTC AGT GTC TCC A-3' |
|  | Reverse | 5'-CTC CGT TTC CAG AAT ACA CAC A-3' |
| *Arl13b* | Forward | 5'-TCA GGA AAG CCT ATA TTG GTG CT-3' |
|  | Reverse | 5'-AGG CAC TTG TGC TCG TTG AC-3' |
| *Cdk2* | Forward | 5'-CCT GCT TAT CAA TGC AGA GGG-3' |
|  | Reverse | 5'-TGC GGG TCA CCA TTT CAG C-3' |
| *Cyclin A2* | Forward | 5'-TGG ATG GCA GTT TTG AAT CAC C-3' |
|  | Reverse | 5'-CCC TAA GGT ACG TGT GAA TGT C-3' |
| *Pparγ* | Forward | 5'-TGG CAA AGC ATT TGT ATG ACT C-3' |
|  | Reverse | 5'-ATT TGT CCG TTG TCT TTC CTG T-3' |
| *Cebp-α* | Forward | 5'-AGA AGT CGG TGG ACA AGA ACA G-3' |
|  | Reverse | 5'-GTT GCG TTG TTT GGC TTT ATC T-3' |
| *Srebp-1* | Forward | 5'-CTA GAG CGA GCG TTG AAC TGT A-3' |
|  | Reverse | 5'-TAT CCA AGG GCA TCT GAG AAC T-3' |
| *Fasn* | Forward | 5'-CTC TGG TGG TAT CCA CAT CTC A-3' |
|  | Reverse | 5'-GTG AAA ACG AAC TTT TCC AAG G-3' |
